# Editorial: Frontiers of Sulfur Metabolism in Plant Growth, Development, and Stress Response

**DOI:** 10.3389/fpls.2015.01220

**Published:** 2016-01-12

**Authors:** Stanislav Kopriva, Dibyendu Talukdar, Hideki Takahashi, Rüdiger Hell, Agnieszka Sirko, Stanislaus F. D'Souza, Tulika Talukdar

**Affiliations:** ^1^Botanical Institute and Cluster of Excellence on Plant Sciences (CEPLAS), University of CologneCologne, Germany; ^2^Department of Botany, Raja Peary Mohan College, University of CalcuttaUttarpara, India; ^3^Department of Biochemistry and Molecular Biology, Michigan State UniversityEast Lansing, MI, USA; ^4^Centre for Organismal Studies, University of HeidelbergHeidelberg, Germany; ^5^Department of Plant Biochemistry, Institute of Biochemistry and Biophysics, Polish Academy of SciencesWarsaw, Poland; ^6^BMG Bhabha Atomic Research CentreMumbai, India; ^7^Department of Botany, Acharya Prafulla Chandra Roy Government CollegeSiliguri, India

**Keywords:** sulfur

Plants assimilate inorganic sulfur and metabolize it further to organic sulfur compounds essential for plant growth, development, and stress mitigation. Animals including humans in turn depend on plants and microorganisms providing these essential compounds, such as the amino acid methionine, which they cannot synthesize. Furthermore, a number of sulfur-containing metabolites provide the characteristic tastes and smells of our food, and many of them are known to have health promoting and protective properties. Thus, adequate supply of sulfur can be a critical factor affecting crop yield and production of beneficial phytochemicals. However, because of the reduction in anthropogenic emission of sulfur dioxide to the atmosphere, particularly from developed countries, sulfur deficiency has become a problem for agriculture and in many areas sulfur fertilization is required to ensure yield, quality, and health of crops. Such an impact of sulfur has triggered research into mechanisms of sulfur metabolism in plants and its regulation. Indeed great progress has been made over the last decades as summarized in several recent reviews (Takahashi et al., 2011; Sauter et al., 2013; Calderwood and Kopriva, 2014). Starting with identification of genes encoding components of sulfur metabolism, research in molecular biology and molecular genetics has brought us toward finding regulators and signals controlling the pathway (Maruyama-Nakashita et al., 2006; Gigolashvili et al., 2007; Hirai et al., 2007), and describing natural variation in diverse sulfur related traits (Kliebenstein et al., 2001; Loudet et al., 2007; Chao et al., 2014). In addition, questions related to regulation of sulfur metabolism have been on the forefront of systems biology (Maruyama-Nakashita et al., 2003; Hirai et al., 2005; Nikiforova et al., 2005) and quantitative genetics (Loudet et al., 2007). This research topic organized in *Frontiers in Plant Science* has been an opportunity to present our current understanding and research progress focused on a number of interesting aspects in plant sulfur metabolism. We aimed to cover broad research topics in sulfur nutrition and metabolism by compiling diverse types of articles: original research reports to exemplify new information on questions the sulfur research community is addressing, focused reviews to provide detailed updates to specific topics, and perspectives to review a progress but also to address the questions for the next decade(s) of research. This concept found indeed a great support in the sulfur research community with 34 articles contributed by scholars representing wide disciplinary areas.

The original articles span a number of topics, plant species, and methodological approaches. A large number of contributions were focused on the model plant *Arabidopsis thaliana* both using targeted and global approaches. Bohrer et al. clarified one of the long standing questions of sulfate assimilation in Arabidopsis, the genetic identity of cytosolic ATP sulfurylase (ATPS) activity. The authors showed that ATPS2 is the only isoform expressed in the cytosol and described the mechanism of the dual targeting of this protein. Frerigmann and Gigolashvili dissected the interplay of transcription factors in repression of glucosinolate synthesis in response to sulfur starvation, in order to explain previous counterintuitive results. Speiser et al. demonstrated the importance of plastidic cysteine synthesis for acclimation to high light. Laureano-Marín et al. then showed a ubiquitous expression of the major enzyme producing hydrogen sulfide, L-cysteine desulfhydrase, and its repression by auxin. Two other teams used omics tools to answer their research questions. Trentin et al. employed proteomics to show that presence of GGT1 affects apoplastic proteome composition upon UV-B radiation. A transcriptomics and metabolomics analysis of sulfate starvation response and the effects of sulfate resupply by Bielecka et al. resulted in identification of 21 transcription factors potentially controlling the response to sulfur.

However, given the general importance of sulfur for plants, the sulfur research has traditionally involved different plant species, including crops. Several papers thus addressed the effects of sulfur availability on the crop with the highest demand for sulfur, oilseed rape. Weese et al. described the large natural variation in response of *Brassica napus* cultivars to sulfate deficiency. Girondé et al. addressed the response of oilseed rape to sulfate deficiency and demonstrated the importance of remobilization of sulfate from vegetative tissues to reproductive organs. Aghajanzadeh et al. added another piece into the mosaic of sulfate starvation response by showing that glucosinolates do not serve as sulfur storage during sulfate deficiency in young seedlings of *Brassica rapa* and *B. oleracea*. Two articles targeted an old aim of sulfur research, the enhancement of content of S-containing amino acids in plant proteins. Kim et al. found that sulfur supply is the main driver for accumulation of sulfur-rich proteins in soybean. Similarly, Pandurangan et al. demonstrated that sulfur supply rather than genetic modification of protein composition affects the methionine content in common bean.

Also other articles demonstrate the results of sulfur-related research in other species than Arabidopsis. Pégeot et al. focused on a family of glutathione transferases in poplar, compared their expression profiles and identified the substrate specificity of the GSTF1 member of the family. Tavares et al. provided comprehensive analysis of the serine acetyltransferase family in *Vitis vinifera*. Some questions cannot be addressed by the model plant at all, because they concern species-specific metabolism or study processes lacking in Arabidopsis, such as mycorrhiza formation. Thus, Yoshimoto et al. made an important step in understanding of synthesis of organosulfur compounds in garlic, by identification of a γ-glutamyl transpeptidase acting on alliin biosynthetic intermediate, γ-glutamyl-S-allyl-L-cysteine. Schiavon et al. addressed the mechanisms underlying selenium hyperaccumulation of some plant species. They could show that the hyperaccumulator *Stanleya pinnata* possesses a sulfate transporter with a high affinity for selenate and a higher expression of sulfate transporters and genes involved in sulfate assimilation. Maniou et al. described in detail aerenchym formation in sulfur starved maize organs. Sato et al. investigated triacylglycerol synthesis in nutrient starved green alga *Chlamydomonas reinhardtii*, showing that this acclimation process is under control of regulators of sulfate starvation response. Last but not least, Chorianopoulou et al. described how in maize mycorrhiza symbiosis alters the expression patterns of genes involved in iron acquisition. Why is such research part of a sulfur research topic? The precursor of phytosiderophores essential for the iron uptake is the S-containing amino acid, methionine.

The focused reviews allowed detailed updates of current understanding of specific topics, from small gene families to complex processes. Gallardo et al. reviewed a family of sulfate transporters, specifically their roles in the response to drought and salinity. Prioretti et al. moved to the next step in sulfate metabolism and highlighted the diversity of ATP sulfurylases in photosynthetic organisms. Anjum et al. also turned to this gene family and described what is known about the role of ATP sulfurylase in plant stress tolerance. Hirschmann et al. provided a comprehensive review of a family of enzymes involved in secondary sulfur metabolism, the sulfotransferases. Wawrzyñska and Sirko concentrated on the key regulator of sulfate starvation response, SLIM1, and other members of the EIN3-like family of transcription factors, highlighting their similarities, potential interplay in signaling pathways and pointing out the unanswered questions to be addressed by future research. Sirko et al. gave the first overview of a family of *LSU* genes induced by sulfate deficiency and encoding the small proteins with unknown functions. The authors show that these proteins are important for adequate plant response to stress (including sulfur deficiency) and propose that they might have auxiliary function in proteostasis (modulation of the stability) of some yet unidentified protein targets in stress conditions. The role of compartmentation of glutathione in response to stress was addressed by Zechmann. Considine and Foyer focused on the physiological and metabolic responses of grapevine to sulfur dioxide. Gahan and Schmalenberger introduced the world of plant symbiosis with mycorrhiza and rhizosphere bacteria and pointed out the importance of microorganisms for plant sulfur nutrition.

The advantage of the *Frontiers* research topic is the opportunity to publish perspective papers with an objective of addressing the future direction of the research areas. In this topic, several contributions fall into this category. Anjum et al. provided a testable hypothesis of the mechanisms by which glutathione and proline interplay in protecting plants against metal and salinity stress. Bohrer et al. used the recent data on subcellular localization of ATP sulfurylase, adenosine 5′-phosphosulfate (APS) kinase and 3′-phosphoadenosine 5′-phosphosulfate (PAPS) transporter to speculate on the role of APS and 3′-phosphoadenosine 5′-phosphate (PAP) in regulation of the pathway and on the control of sulfur fluxes in the plant. Regulatory mechanisms and sulfur sensing were the topic of Zheng et al. based on their previous finding of a possible transceptor role of sulfate transporter SULTR1;2. Weckopp and Kopriva used transcriptome data from C4 plants to speculate on the connection between sulfur metabolism and C4 photosynthesis. Bloem et al. connected the past with the future, summing up the milestones of research into the connection of sulfur nutrition and crop health—the sulfur induced resistance—and providing an outline of future directions. In a similar concept, Koprivova and Kopriva reviewed current knowledge of molecular mechanisms of regulation of sulfate assimilation and formulated the major open questions. Calderwood et al. then discussed and proposed various mathematical approaches to dissect the control of sulfur fluxes in plants.

Altogether, the research topic as presented here documents recent advances in sulfur research, in fundamental science, as well as applied aspects. The papers compiled in this e-book clearly demonstrate that sulfur research is at the forefront of plant science. The number of knowledge-based questions and challenges identified and listed in individual papers guarantee exciting future of this research topic.

## Conflict of interest statement

The authors declare that the research was conducted in the absence of any commercial or financial relationships that could be construed as a potential conflict of interest.
